# The 17^th^ SSNN International Summer School of Neurology 2022 – Part 1

**DOI:** 10.25122/jml-2022-1023

**Published:** 2022-08

**Authors:** Stefana-Andrada Dobran, Alexandra-Mihaela Gherman, Dafin Fior Muresanu

**Affiliations:** 1 RoNeuro Institute for Neurological Research and Diagnostic, Cluj-Napoca, Romania; 2 Sociology Department, Babes-Bolyai University, Cluj-Napoca, Romania; 3 Department of Neuroscience, Iuliu Hatieganu University of Medicine and Pharmacy, Cluj-Napoca, Romania

**The 17^th^ International Summer School of Neurology organized by the** Foundation of the Society for the Study of Neuroprotection and Neuroplasticity (SSNN) took place between 8–10 July 2022 and was organized as a hybrid event. The aforementioned educational event together with the 5^th^ Teaching Course on Rare Neurological Diseases brought together a lively audience of over 1400 participants in a hybrid format, both online and on-site.

The first day of the Summer School included presentations on the latest and most exciting or pressing issues in Neurology.

The event started with a welcome address by Dafin Muresanu (Romania), Natan Bornstein (Israel), Volker Homberg (Germany), and Wolfgang Grisold (Austria).

Followingly, **Wolfgang Grisold** held a short presentation on the World Federation of Neurology (WFN) institution and activities. The World Federation of Neurology encompasses 6 regions and 123 member societies worldwide. As the World Health Organization approved the Global Action Plan to address epilepsy and other neurological disorders, the 10 years intersectoral action plan of the WFN was addressed, which is set to target:

- Policy;
- Diagnosis and treatment;
- The implementation of strategies for promotion and prevention;
- Fostering research and innovation;
- Strengthening of public health approaches.

The WFN activities include communication with regional and member societies, increasing the impact of international cooperation, education programs, development of publications, and international adaptation.

Volker Homberg (Germany) and Dafin Muresanu (Romania) introduced the first session and covered topics such as neurorehabilitation, post-stroke deficits and post-stroke recovery.

**Volker Homberg (Germany)**, President-Elect of the World Federation of Neurorehabilitation (WFNR), presented a future perspective for neurorehabilitation, also highlighting the pillars of WFNR, namely (1) improvement of science, (2) improvement of services, and (3) improvement of education in neurorehabilitation. In the context of the global situation, where over 1 billion people live with some form of disability (and the number is increasing), prof. Homberg highlighted the differences in the availability of trained staff among different income countries and discussed high-tech and low-tech options for neurorehabilitation. Prof. Homberg pinpointed the strategies for neurological rehabilitation (restoration *vs*. compensation): replacement of dead tissue, “wake up” alternative structures, teaching bypass strategies, replacing functions by aiding devices, and adapting the environment to patients. He presented exciting avenues of technology for rehabilitation, such as rehabilitation robots, VR/AR technology, and brain-computer interfaces, putting an emphasis on the role of motivation in neurorehabilitation and the prospect of serious games, which can have a decisive role in therapeutic strategies. In contrast, the importance of more available low-tech options and the role of enriched environments were also marked, and the limitations and opportunities for both high-tech and low-tech undertakings were discussed. Pharmacological options for recovery and the latest achievements in neurorehabilitation were also addressed.

**Dafin Muresanu (Romania)**, Chairman at the Department of Neurosciences, Iuliu Hatieganu University of Medicine and Pharmacy in Cluj-Napoca (Romania), presented advances and updates in neurorecovery after stroke, pinpointing the need for better evidence-based approaches in stroke, supported by the following 3 pillars:

1. Theory and basic research;
2. Evidence-based parameters;
3. External validity.

According to Prof. Muresanu, the whole science and practice of neurorecovery are based on the pillars above. The burden of neurological disorders in Europe was also discussed, with stroke being a significant component of the disease burden. Prof. Muresanu showcased the AHA/ASA guidelines, the role and advantages of the GRADE system, as well as the similarities with EAN guidelines. The role of multimodal agents in contrast to neuroprotectants for neurorehabilitation was also approached.

The last presentation of the session was held by **Michel Brainin (Austria)**, Emeritus Professor of Neurology and Chair at the Department of Clinical Neurosciences and Preventive Medicine of Danube University in Krems (Austria), centered on cognitive deficits after stroke. Prof. Brainin's first message was that the most common manifestation of vascular diseases is not reflected in stroke but in cognitive impairment and dementia. Furthermore, he took a more focused glance at vascular cognitive impairment and what cognitive domains it affects. He discussed the risk and protective factors for dementia and stroke, presenting a case study. He also approached several topics, such as small vessel disease, strategic infarct, preexisting vascular damage, and preexisting Alzheimer's disease.

The second session was introduced and coordinated by Michael Brainin (Austria) and Natan Bornstein (Israel).

The first to present was **Wolfgang Grisold (Austria)**, President of WFN, who discussed the educational activities of the World Federation of Neurology (WFN). Prof. Grisold discussed the IGAP (Intersectoral Global Action Plan on Epilepsy and Other Neurological Disorders), which centers around developing education training, structures for training, making research attractive, and promoting a public health perspective. He then presented the mission and current WFN educational activities, including accredited training centers, department visit programs, junior travelling fellowships, WFN grants, eLearningHub, and Young Neurologist programs.

**Johannes Vester (Germany)**, Head of Biometry & Clinical Research at the Institute for Data Analysis and Study Planning (IDV) in Gauting (Germany) then presented “A new gold standard to improve TBI clinical research – the multidimensional approach”. The gaps in traditional trial designs and the need for multidimensional approaches were highlighted to capture the global status of TBI patients as accurately and comprehensively as possible. He outlined current approaches and different scales used in research, pinpointing the more accurate picture of outcomes that the multidimensional analysis offers in contrast to the often employed one-criterion paradigm, which has been the standard in the research of neuroprotective treatments over the past decade. To support his point, he discussed the meta-analysis of the CAPTAIN trials, trials based on multidimensional approaches.

**David Vodusek (Slovenia)**, Emeritus Professor of Neurology at the Faculty of Medicine from the University of Ljubljana (Slovenia), presented “Neurological and neurophysiological evaluation of the lower sacral segments”. Prof. Vodusek discussed steps in the diagnostics, the difficulty of assessing lesions affecting the peripheral nervous system in contrast to central nervous system (CNS) evaluation, methods, the testing of the motor function along with some issues in reflex testing, diagnosis of neurological lesions in sacral segments through different tools (including electromyography) and many other inspiring subjects.

The 3^rd^ session of the day was introduced by Antonio Federico (Italy) and Tudor Lupescu (Romania), addressing neuropathies and neurosciences in migrant populations.

**Antonio Federico (Italy)**, Emeritus Professor of Neurology at the Department of Medicine, Surgery and Neurosciences from the University of Siena in Siena (Italy) firstly discussed aspects of neurology and neurosciences in migrants and refugees, describing the global situation and the effects of immigration, both positive and negative, and pinpointed the phenomenon of “brain drain” – the migration of highly educated individuals- in certain territories. Furthermore, Prof. Federico outlined the relationship between refugee status and migration with the health system and the aspects which link migration and neurosciences (*e.g*., the variable risk of several common neurological diseases, whether cerebrovascular, degenerative, inflammatory, or other). He discussed the specific brain diseases in immigrants and refugees, showcased the influence of migration on brain development, and outlined some protective factors encountered in migrants which mediate cognitive disturbances. Finally, the needs and limitations of approaching the neurological health of migrants and the impact of migration on children were also topics touched upon.

**Max Hilz (Germany)**, from the Icahn School of Medicine at Mount Sinai, New York (USA) and University Erlangen-Nuremberg (Germany) offered an insightful presentation on “Diabetic autonomic neuropathy”, discussing the complications of diabetes and the relationship of diabetes with neuropathies, highlighting the importance of making an early diagnosis to prevent fatal outcomes. In addition, he discussed the fluctuation of heart rate and blood pressure with age, how to diagnose cardiac diabetic neuropathy and offered recommendations for assessment and diagnostic criteria.

**Tudor Lupescu (Romania)**, the Head of the Neurology Department from Agrippa Ionescu Hospital in Bucharest (Romania), presented diagnostic approaches to neuropathies. Differential diagnostics, profiling, timing assessment, types of neuropathies and their symptoms were the topics covered and presented in an inspiring case study.

The 4^th^ session was introduced by Marc Fisher (USA) and Urs Fischer (Switzerland).

**Marc Fisher (USA)**, Professor of Neurology at Harvard Medical School (USA) showcased his presentation “Repurposing neuroprotection (Cyto) and recovery enhancing drugs in the thrombectomy era”, where he discussed the ischemic penumbra as the target of acute stroke therapy, the relevance of combining cytoprotection with reperfusion, and the need for multi-targeted interventions, as well as the factors for multi-targeted approach. Decompressive surgery was also addressed as a topic. Further on, **Urs Fischer** (Switzerland), the Chairman of the Department of Neurology at the University Hospital in Basel (Switzerland) presented the “Management of spontaneous intracerebral haemorrhage”. Prof. Fischer underlined the need for more recent evidence-based guidance in the global burden of stroke, discussed therapeutic approaches and the importance of lowering blood pressure, and showcased international randomized control trials, presenting their outcomes.

**Natan Bornstein (Israel)**, Professor of Neurology at Tel-Aviv University, Sackler Faculty of Medicine (Israel) discussed “Diabetes and Stroke”. One hundred years after the discovery of insulin, his insightful presentation began with a mention of diabetes as the leading risk factor for stroke and a short reminder of the increasing global burden of stroke (12/2 million new stroke cases per year with a significant proportion of DALYs). It repeatedly highlighted the critical role of primary and tertiary prevention for stroke. Type 2 diabetes will increase the risk of stroke and double the risk of stroke occurrence, stroke outcomes being significantly worse in patients with diabetes. Prof. Bornstein highlighted that diabetic patients have a risk of stroke similar to people without diabetes who are 25 years younger. Furthermore, Prof. Bornstein discussed the role of gut hormones and incretin-based therapies, presenting randomized control studies, and presented the role of new therapies that demonstrated protective effects in patients with type 2 diabetes and higher risk of cardiovascular complications or with established cardiovascular disease (SGLT 2 inhibitors: GLP – 1 RAs). Moreover, he talked about the importance of neurologists in managing patients with type-2 diabetes mellitus, controlling diabetes and other risk factors to prevent initial stroke and stroke occurrence, and the importance of preventing atherosclerosis both for stroke and dementia prevention. He mentioned that controlling risk factors through medication and lifestyle modification can reduce the risk of stroke by 90%. Following his presentation, **Laszlo Csiba (Hungary)**, Professor of the Department of Neurology at the Debrecen University in Debrecen (Hungary) discussed interesting cases from daily practice.

Other information and further details on the 17^th^ International Summer School of Neurology, the “Psychiatry Perspectives in Neurocognitive Disorders” parallel session, and the 5^th^ Teaching Course on Rare Neurological Diseases will be highlighted in future materials.

